# Natural Resources Resistance to Tomato Spotted Wilt Virus (TSWV) in Tomato (*Solanum lycopersicum*)

**DOI:** 10.3390/ijms222010978

**Published:** 2021-10-12

**Authors:** Shiming Qi, Shijie Zhang, Md. Monirul Islam, Ahmed H. El-Sappah, Fei Zhang, Yan Liang

**Affiliations:** 1 College of Horticulture, Northwest A&F University, Xianyang 712100, China; qishiming2008@nwafu.edu.cn (S.Q.); 13289316916zsj@nwafu.edu.cn (S.Z.); monirul@nwafu.edu.cn (M.M.I.); ahmed_elsappah2006@yahoo.com (A.H.E.-S.); feizhang@nwsuaf.edu.cn (F.Z.); 2 State Agriculture Ministry Laboratory of Northwest Horticultural Plant Germplasm Resources & Genetic Improvement, Northwest A&F University, Xianyang 712100, China; 3 Genetics Department, Faculty of Agriculture, Zagazig University, Zagazig 44511, Egypt

**Keywords:** tomato, tomato spotted wilt virus, resistance gene, resistant resource, molecular marker-associated breeding

## Abstract

Tomato spotted wilt virus (TSWV) is one of the most destructive diseases affecting tomato (*Solanum lycopersicum*) cultivation and production worldwide. As defenses against TSWV, natural resistance genes have been identified in tomato, including *Sw-1a*, *Sw-1b*, *sw-2*, *sw-3*, *sw-4*, *Sw-5*, *Sw-6*, and *Sw-7*. However, only *Sw-5* exhibits a high level of resistance to the TSWV. Thus, it has been cloned and widely used in the breeding of tomato with resistance to the disease. Due to the global spread of TSWV, resistance induced by *Sw-5* decreases over time and can be overcome or broken by a high concentration of TSWV. How to utilize other resistance genes and identify novel resistance resources are key approaches for breeding tomato with resistance to TSWV. In this review, the characteristics of natural resistance genes, natural resistance resources, molecular markers for assisted selection, and methods for evaluating resistance to TSWV are summarized. The aim is to provide a theoretical basis for identifying, utilizing resistance genes, and developing tomato varieties that are resistant to TSWV.

## 1. Introduction

Tomato (*Solanum lycopersicum*) is one of the most important economic vegetable crops. As a major producer and exporter of tomato products the worldwide, China has over one million hectares of harvested area and a total tomato production of ~63 million tonnes in 2019. These values are the highest in the world [1]. Disease is the major biotic stress in tomato production and quality. A total of 136 viral species severely harm tomatoes [2], and the Tomato spotted wilt virus (TSWV) is one of the most harmful.

**Figure 1:**
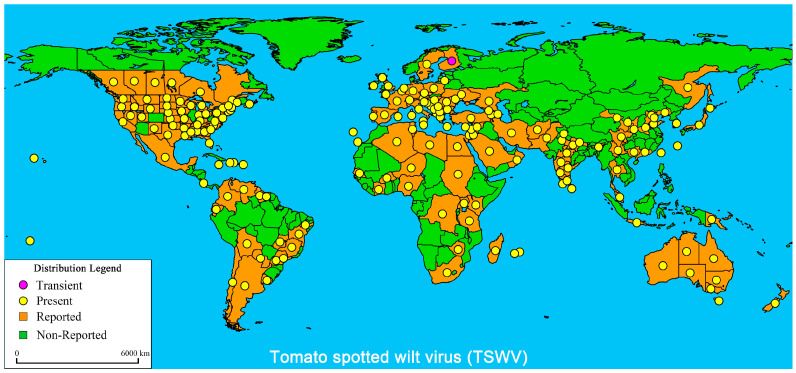
Global distribution of Tomato spotted wilt virus (TSWV). The global distribution of TSWV disease published by the EPPO Global Database (20 May 2021) and modified about China distribution [21,22,23,24]. The purple marks indicate transient infection, yellow marks indicate present infection, orange marks indicate reported infection, and green marks indicate non-reported infection.

The TSWV belongs to the species *Tospovirus*, the genus *Orthotospovirus*, the family *Tospovirdae*, and the order *Bunyaviridae* [3]. It is the only species of RNA globular virus species that infects plants [4]. The TSWV is listed as one of the top 10 most important plants viruses worldwide [5]. The TSWV virions are oblate, easily deformed, and have an envelope structures and continuous protrusion layers on their outer membranes. The genome contains three different genomic RNA strands: Large (L) RNA, Medium (M) RNA, and Small (S) RNA, which encode five proteins. The L RNA is a negative-sense RNA that encodes RNA-dependent RNA polymerase (RdRp) [6], has a replication-related protein function, and can work together with the encoding factors of hosts [7,8]. M RNA is a double-sense and antisense RNA encoding protein in the amino and carboxy-terminal positions within the glycoprotein precursors (G_n_G_c_), which play crucial roles in virion assembly, maturation, and release in a host organism [9,10]. M RNA is a sense RNA encoding viral non-structural proteins (NSm) and mainly promotes TSWV infection [11]. S RNA is a double-sense and antisense RNA that encodes nucleocapsid proteins (N) and a sense RNA that encodes non-structural proteins (NSs) [12]; both types of proteins play crucial roles in the TSWV infection cycle [13,14,15]. The TSWV has an extremely wide range of susceptible hosts, including many important agricultural and field crops (infecting more than 1090 plant species), especially tomato, pepper, potato, and tobacco [16,17,18,19,20]. TSWV diseases are widely distributed, and the most severe cases are found in temperate and subtropical areas (Figure 1).

The rapid spread of *Frankliniella occidentalis* (Western flower thrips) carrying the TSWV has seriously harmed tomato cultivation and production. The TSWV reduces yield in large areas and the marketable value of tomato or even causes the death of tomato plants [25,26]. Numerous tomato plants (such as those that carry the *Sw-5* gene) resistant to the TSWV have been screened, and many excellent reviews on the different aspects of TSWV biology, thrips vector-mediated transmission, resistance strategies, and plants’ innate immunity to the TSWV have been published [10,27,28,29,30,31,32,33]. In this study, a systematic overview of global distribution, TSWV symptoms, and inoculation method, organized resistance gene defense against the TSWV, marker-assisted selection markers of resistance breeding, and natural resistant resources is provided, and the focus is innate immunity to TSWV. The aim is to provide a basis for exploring genes involved in resistance against the TSWV and for research on disease resistance breeding in tomato.

## 2. TSWV Symptoms in Tomato Plants

**Figure 2:**
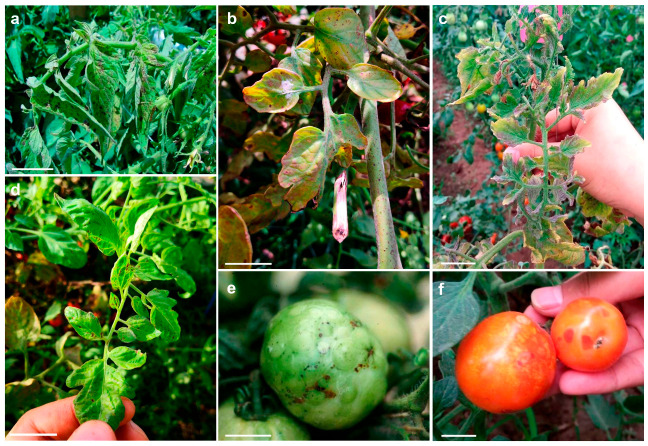
Typical symptoms of Tomato spotted wilt virus (TSWV) in different growth stages of field tomato plants. (**a**,**b**) dark-brown flecks and necrotic spots; (**c**,**d**) turn into copper-colored rolls, purple veins, terminal bud necrosis, and rings on leaves and stems; (**e**) dark-brown flecks, faded necrotic spots and tumor-like protrusions; (**f**) chlorotic and bright yellow rings. The white bar represents 25 mm.

TSWV infection in field tomatoes is systemic, rendering an entire plant susceptible and resulting in yield losses leading to huge economic losses [34]. Infected tomato plants are usually dwarfed and have necrotic streaks and dark-brown flecks on their leaves, stems, and fruits (Figure 2). The first symptoms in tomato seedlings are inhibited growth points and copper-colored rolls of young leaves. Subsequently, many small dark-brown flecks form, and leaf veins become purple (Figure 2a–c). Growth points appear, leaf necrosis and droop occur, and the stem end forms brown necrotic streaks. The plants show inhibited growth or are completely dwarfed or become deciduous and wilted (Figure 2c,d). In the green fruit period, chlorotic rings appear on the fruit, green fruit is slightly raised, the ring is not obvious, faded necrotic spots appear, which are tumor-like protrusions, and the fruits easily falls off (Figure 2e). During the fruit-ripening period, the fruit has red-yellow or red-white ring spots and bright yellow ring markings, particularly the red ripe fruits (Figure 2f). Identifying resistant resources and genes is the first step to solve these problems.

## 3. Methods for Identifying Resistance to TSWV in Tomato

### 3.1. Mechanical Inoculation

Mechanical inoculation is a simple and effective method for conducting genetic research on resistance to TSWV. A large number of plants can be quickly screened simultaneously with several isolates for the identification of resistant resources [35,36]. An inoculation buffer containing potassium phosphate buffer (0.1 M, pH 7.0), sodium sulphite (0.2% *w*/*v*), and polyvinyl pyrolidon (2% *w*/*v*) was used. The inoculum was prepared by mixing 1 g of infected leaf tissue with 10 mL of inoculation buffer and 1% carborundum (600 mesh). The fully expanded leaves of the plants at the four-, five, and six-leaf stages were inoculated by rubbing with a brush or cotton swab on the surfaces of the tomato leaves [37]. The inoculated plants were maintained in an environment-controlled greenhouse at 25 °C (day) or 18 °C (night) and at 60% (day) or 95 % (night) of relative humidity for the monitoring of the symptoms. Symptoms were evaluated once a week after inoculation, and six disease grading criteria were used: Asymptomatic, mild, moderate, severe, and whole plant necrosis [38,39]. The incidence rate and disease index were calculated for the identification of resistant tomato plants.

### 3.2. Thrip Inoculation

Transmission by thrips is difficult to manage, which exposes plants to a high inoculum pressure, and is an extremely effective graft-inoculated method. In general, inoculation needs to be carried out under strictly controlled environmental conditions, which are 25 ± 2 °C temperature, 50% ± 5%/90% ± 5% of relative humidity (day/night), 14 h/10 h (day/night) photoperiod, and cover with anti-thrips mesh (100 mesh). Susceptible plants were inoculated with TSWV isolates at the four-, five, and six-leaf stages and cultivated for symptom development. Then, systemically infected plants were fed to the first instar larvae (0–2 h old) of *Frankliniella occidentalis* for 2 days to carry the TSWV isolates [26]. The viruliferous larvae were cultivated into adults on healthy plants. Then, the viruliferous adult thrips were used in inoculating plants for 48 h. After the inoculation, the symptoms of the plants were systematically monitored once a week, and resistance and susceptibility were investigated.

Mechanical inoculation and transmission through thrip inoculation are the commonly used methods in screening and identifying resistant germplasm sources in tomato. Mechanical inoculation is easier to implement and is a more effective management method, especially in field natural disease identification. However, it has poor repeatability and depends only on a single test result; it can only identify the resistance of a host to a virus without considering host–vector interactions, resulting in the loss of excellent resistant materials [40]. Furthermore, the method is laborious when a large number of test plants are use. Fortunately, Mandal et al. [41] developed a rapid and efficient pressurized spray inoculation method for the TSWV, but the method seems to be costly and is not widely used. By contrast, transmission by thrips is the closest inoculation method for identifying vector-mediated TSWV resistance components for natural infection. However, this method requires the feeding of viruliferous thrips, and high control on the experimental conditions is required for transmission. However, whether the results pertain to TSWV resistance, thrips resistance, or both is unclear. Mechanical inoculation facilitates the identification of the effects of virus replication and migration, and thrips inoculation facilitates the study of the impact of materials on thrip feeding behavior [42]. Therefore, combining the two methods for identifying plants with different resistance mechanisms according to their complementary information prevents the potential loss of resistance resources [35].

## 4. Natural Resources Resistant to the TSWV in Tomato

Through the efforts of tomato breeders in the past decades, natural TSWV-resistant germplasm resources have been screened and identified in many tomato lines, genotypes, and cultivars. They are distributed in cultivated and wild tomatoes and mainly distributed in *Lycopersicon peruvianum* Mill., *Lycopersicon chilense* Dun., *Lycopersicon pimpinellifolium* Mill., and other wild tomatoes. Several TSWV resistance genes have been identified in these resources (Table 1). Many germplasm resources of resistance have been discovered from the Porter’s strain of *L. pimpinellifolium* since 1945. Subsequently, a highly resistant cultivar was detected in the cultivar ‘Stevens’ from *S. peruvianum* and the LA 1938 from *L. chilense* [43]. Some plants show high resistance, such as ‘Stevens’, ‘Viradora’, LA0370, LA0445, LA0446, LA2581, LA4445, PE-18/UPV-1, and RDD from *L. peruvianum* and the LA 1938 from *L. chilense*. The preferred and safest method for combating TSWV is identifying novel resistant plants. An extensive and in-depth evaluation of tomato plants for which resistance has not been determined is important.

## 5. Natural Genes Resistant to TSWV in Tomato

Eight loci, namely, *Sw-1a*, *Sw-1b*, *sw-2*, *sw-3*, *sw-4*, *Sw-5*, *Sw-6*, and *Sw-7*, for resistance to TSWV have been discovered in the different materials of tomatoes. They originate from cultivated and wild tomatoes, and only *Sw-5* has been cloned because of its effective resistance to the TSWV [50,62]. *Sw-1a*, *Sw-1b*, *sw-2*, *sw-3*, *sw-4*, and *Sw-6* exhibit some degree of resistance to specific TSWV [45,63,64]. As a newly discovered gene in recent years, *Sw-7* has a small region range and offers resistance to a wide range of TSWV [65,66,67].

### 5.1. Sw-1a and Sw-1b

The *Sw-1* gene contains two gene clusters, *Sw-1a* and *Sw-1b*, and a single dominant and allele pair. Finlay [44] demonstrated that *Sw-1a* and *Sw-1b* genes are present in the Pearl Harbor and Porter’s strains of *L. pimpinellifolium*, and Rey de los Tempranos and Manzana varieties of *Lycopersicon esculentum*, respectively. The tomato cultivar PI 128657 from *L. peruvianum* has the *Sw-1* gene that resists the isolate TSWV6 [58,68]. However, the isolate specificity and effective resistance of the two gene clusters are limited, particularly in terms of regulating the reactions to the TSWV strains TB2 (tip blight 2), N1 (necrotic), and R1 (ringspot), and TB3 (tip blight 3) [44]. The clusters have been overcome by various TSWV isolates and other tospoviruses [63,64]. Furthermore, the molecular mechanisms and chromosomal locations of the genes are unknown, and thus the genes are rarely used in tomato breeding.

### 5.2. Sw-2, Sw-3, and Sw-4

Three recessive genes, namely, *sw-2*, *sw-3*, and *sw-4*, for TSWV resistance in tomato have been discovered, which appear to be inherited independently, and come from the Porter’s and Pearl Harbor strains of *L. pimpinellifolium*, and Rey de los Tempranos and Manzana varieties of *L. esculentum* [44]. However, these resistance genes, which are isolate specific for TSWV, have been quickly overcome [63,64]. As recessive genes, the genes should show specific resistance in homozygotes. This genetic configuration restricts the development of hybrids [53]. Thus, the genes have not been utilized in commercial breeding.

### 5.3. Sw-5

**Figure 3:**
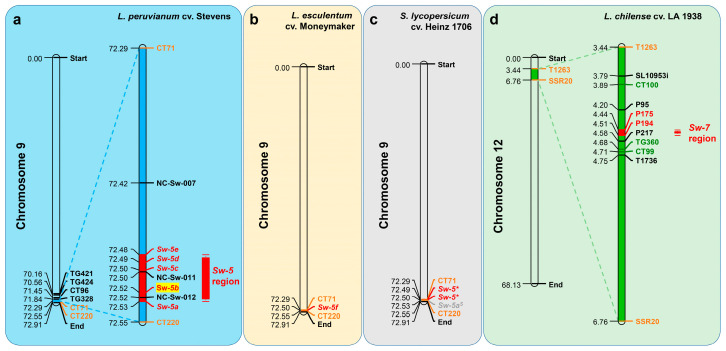
Mapping of TSWV resistance genes on tomato chromosomes. (**a**) The site of *Sw-5* (*Sw-5a*, *Sw-5b*, *Sw-5c*, *Sw-5d*, and *Sw-5e*) on chromosome 9 of *L. peruvianum* cv. Stevens. The red boxes represent the narrowest area where the *Sw-5* cluster region is located [50]; (**b**) the site of *Sw-5f* on chromosome 9 of *L. esculentum* cv. Moneymaker; (**c**) the site of Sw-5*, *Sw-5a^S^* on chromosome 9 of *Solanum lycopersicum* cv. Heinz 1706; (**d**) the site of *Sw-7* region on chromosome 12 of *L. chilense* cv. LA 1938. The green boxes represent the narrowest area where the *Sw-7* region is currently located. The orange-yellow font markers (T1263 and SSR20) represent the markers mapped by Stevens [72] and Dockter et al. [66]. Green font markers (CT100 and TG360) and red font markers (P175 and P194) represent the markers mapped by Scott et al. [67].

*Sw-5* is a single dominant quality resistance gene responsible for resistance to a broad range of tospovirus species [64]. Originating from *L. peruvianum*, this gene has been identified and introgressed in the fresh market tomato cultivar (*Lycopersicon esculentum*) Stevens [63] and has been mapped near the telomeric region of the long arm in chromosome 9 between the RFLP markers CT71 and CT220 [62]. It is closely linked to the CT220 marker (within 65 kb) [50,51,69]. The *Sw-5* locus is a member of a loosely clustered gene family and contains six homologous paralog genes: *Sw-5a*, *Sw-5b*, *Sw-5c*, *Sw-5d*, *Sw-5e*, and *Sw-5f* [31,62]. *Sw-5a*, *Sw-5b*, *Sw-5c*, *Sw-5d*, and *Sw-5e* have been cloned in *L. peruvianum* cv. ‘Stevens’, and *Sw-5f* has been cloned in *L. esculentum* ‘Moneymaker’ (Figure 3a,b) [60]. Sw-5* is highly conserved *Sw-5a* and *Sw-5b*, and *Sw-5a^S^* are highly conserved *Sw-5c*, *Sw-5d*, and *Sw-5e* according to the sequencing and annotation of the tomato genome (*S. lycopersicum* cv. Heinz 1706) in susceptible tomatoes and have been functionally studied (Figure 3c) [28,70,71].

*Sw-5a* and *Sw-5b* genes are highly homologous (95%). However, only *Sw-5b* has broad-spectrum resistance to distinct TSWV isolates [45,63] and is a key gene in resistance to the TSWV [51]. *Sw-5b* mediates resistance to the related tospovirus species, tomato chlorotic spot virus (TCSV), tomato zonate spot virus (TZSV), and groundnut ring spot virus (GRSV) [51,52,64] and even to the less related impatiens necrotic spot virus (INSV) [73,74]. The roles of the *Sw-5c*, *Sw-5d*, and *Sw-5e* genes in resistance to TSWV and related resistance and molecular mechanisms need further study.

**Figure 4:**
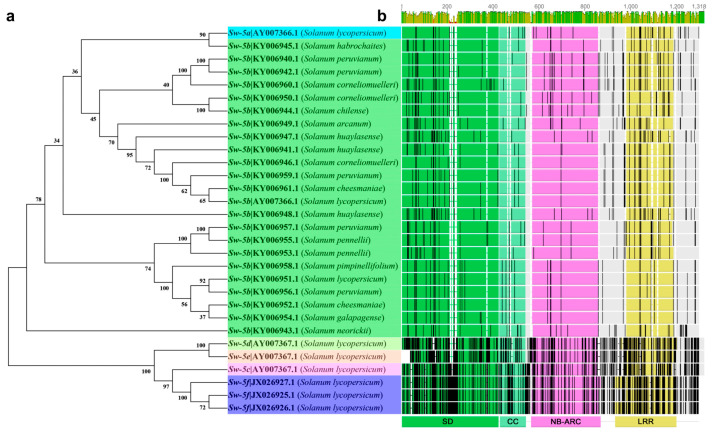
Cluster and topology analysis of the *Sw-5* gene cluster and orthologs from different tomatoes. (**a**) Cluster analysis based on the *Sw-5* gene cluster DNA sequence; (**b**) alignment and topology analysis based on the Sw-5 protein cluster, an overview of the roles of the amino-terminal *Solanaceae* domain (SD) and coiled-coil (CC), central nucleotide-binding adaptor shared by apoptotic protease-activating factor-1 (APAF-1), R proteins, and a CED-4 (NB-ARC); and carboxy-terminal leucine-rich repeat domains (LRR) in the Sw-5 protein cluster. The black vertical lines indicate an amino acid difference, and gray blocks indicate the same amino acid sequence.

The Sw-5 clustered proteins are members of the resistance (R) gene family and encoded by the amino-terminal *Solanaceae* domain (SD) and coiled-coil domain (CC) domain; central nucleotide binding-adapter shared by APAF-1, R proteins, and a CED-4 (NB-ARC); and a leucine-rich repeat (LRR) domain (Figure 4b) [50,51,71,75]. Sw-5b is a typical CC-NB-ARC protein. The main reason that Sw-5b has broad-spectrum resistance is that its SD domain can specifically recognize a conserved 21-aa (amino acid) in the TSWV NSm. The NSm region is highly conserved in American-type tospoviruses, but not in Euro-Asian-type tospoviruses [47,76]. The *Sw-5b* genes have been found in different tomato germplasm materials through the sequencing of the tomato genome [47]. Sequence differences among the genes of different tomato plants are extremely large (Figure 4a,b). Although *Sw-5* is widely used in tomato resistance crossbreeding, it is not completely immune to the TSWV. It will be overcome in the presence of a high concentration of TSWV pressure or stable resistance-breaking isolates [77]. The surface of the fruit has ring spots, and the leaves show necrosis [78,79]. Its resistance is limited [80,81].

### 5.4. Sw-6

A single TSWV resistance gene *Sw-6* from *L. peruvianum* is identified in the *L. esculentum* introgressed UPV-32 line (Table 1) [53]. The gene is resistant to typical TSWV isolates (e.g., T-941117 and HA-931100 Spanish isolates). However, resistance cannot be identified when screening is performed under greenhouse conditions [82]. The resistance of the *Sw-6* locus confers partial resistance, is not as strong that provided by *Sw-5*, is inherited independent of *Sw-5* and the UPV 1 resistance gene (introgressed from *L. peruvianum*) [45]. It exhibits partial resistance or incomplete dominance when thrip (*Frankliniella occidentalis*) inoculation is performed by TSWV isolate aggressiveness, and it is effectivity range is narrower than the effectivity ranges of *Sw-5* and the UPV 1 resistance gene due to its incomplete penetration and gene dosage effects [45]. Nevertheless, although the TSWV partially conditions *Sw-6* to isolate aggressiveness, the resistance of the gene differs from that of the *Sw-1a*, *Sw-1b*, *sw-2*, *sw-3*, and *sw-4* [44,45]. Unfortunately, the molecular mechanism of the *Sw-6* resistance gene has not been characterized. Determining whether these genes represent different genes located on distinct chromosomes or on the same chromosomes, and whether they represent different alleles and dosages from a well-known resistance gene cluster, remain unclear [28]. Thus, the resistance levels and persistent resistance in tomato can be improved by facilitating the crossing of the *Sw-6* gene with other resistance genes, [68]. The gene plays a positive role in the breeding of disease-resistant tomato.

### 5.5. Sw-7

*Sw-7* is a single dominant quality gene that confers field resistance against various TSWV isolates [43,83,84]. It is derived from the breeding material Y118 (Flag 925-2) selected with *S. chilense* accession LA 1938 (Table 1) and generally resides between markers T1263 (45.0 cM) and SSR20 (58.2 cM) on chromosome 12 [66,72,82,85]. The region is narrowed between P175 (4.44 Mb) and P194 (4.51 Mb) (Figure 3d) [67]. However, this locus has not been mapped and cloned, and the specific molecular mechanism is unknown.

*Sw-7* is not linked to *Sw-5* [83], but provides field resistance to the various isolates of the TSWV in Florida, Georgia, Hawaii, and South Africa [86]. Greenhouse utilizing trials are resistant to isolates that overcome tomatoes that are homozygous for *Sw-5*, and it shows a resistance mechanism different from that of *Sw-5* [65,66]. Therefore, *Sw-7* was used as an alternative locus conferring resistance to a wide range of TSWV strains. The *S. chilense*-based germplasm has been promoted in Australia, Thailand, Taiwan, and Italy [86]. Researchers performed a comprehensive transcriptome profiling, functional characterization using an *Sw-7* nearly-isogenic line and a TSWV-susceptible parent (Fla.8059) upon inoculation with the TSWV showed the potential involvement of the pathogenesis-related protein 5 (PR-5) in *Sw-7* resistance. It is associated with *Sw-7* resistance [37,87]. *Sw-7* resistance effectively facilitates the breeding of disease-resistant tomatoes and serves as a source of resistance germplasm that provides protection against the TSWV. For the identification of resistance genes for tomato breeding, molecular markers associated with resistance should be developed.

## 6. Molecular Markers for Resistance to TSWV in Tomato

Due to the geographical specificity of the TSWV isolates distributed with geographical areas, the identification results of natural field inoculation are not reproducible. The disease is limited by many factors, such as environmental influences, which causes great uncertainty for the identification of TSWV-resistant tomato materials. However, in marker-assisted selection (MAS), molecular markers are closely linked to the genes that determine target traits, the desired gene is detected with molecular markers, and target traits are selected. MAS has the advantages of reducing breeding costs and improving breeding selection accuracy and is not affected by environmental conditions. Tomato is considered a model plant for commercial breeding using molecular markers [88]. The development of molecular linkage markers for TSWV resistance genes is mainly focused on the research of *Sw-5* and *Sw-7* markers. The linkages molecular markers of *Sw-1a*, *Sw-1b*, *sw-2*, *sw-3*, *sw-4*, and *Sw-6* have not been reported. The linked molecular markers used in resistance breeding against TSWV are summarized in Table 2. The use of these markers has a wide and effective application prospect in the selection and identification of resistant tomato materials, discovery of novel TSWV resistance genes, and acceleration of the breeding processes of tomatoes with TSWV resistance.

## 7. Mechanism of Natural Resistance to the TSWV in Tomato

**Figure 5:**
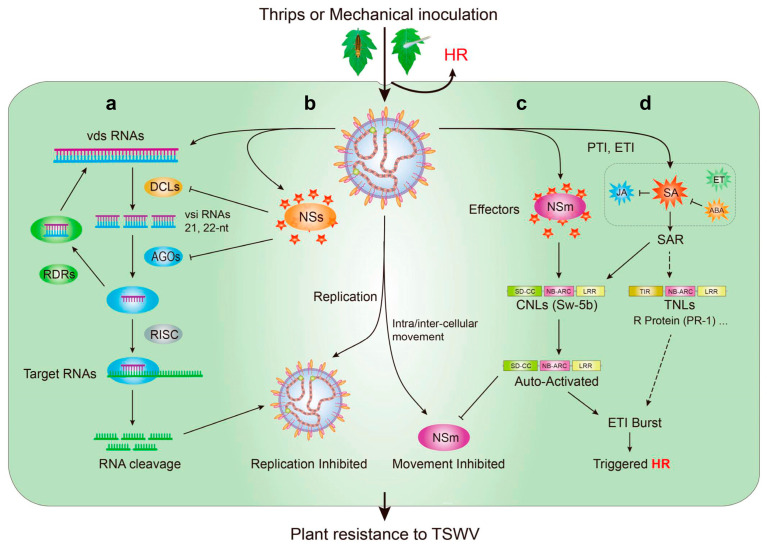
Mechanism with plant innate immunity against Tomato spotted wilt virus (TSWV). (**a**) TSWV invaded by thrips or mechanical inoculation, and depend on the plant cellular machineries to complete their life; (**b**) RNA interference (RNAi) with plant innate immunity is triggered to prevent TSWV invasion, viral dsRNA (vds RNAs) from viral mRNA of TSWV is cleaved into 21, 22-nt viral small interfering RNAs (vsi RNAs) by the RNAse III Dicer-like proteins (DCLs); the vsiRNAs into Argonaute (AGO) activates the RNA-induced silencing complex (RISC) and activates the complementarity and cleavage of viral target RNAs; (**c**) to further combat TSWV invasion, the CNLs (including Sw-5b protein) from tomato sense the effectors NSm and NSs, robust effector-triggered immunity (ETI); and produce the hypersensitive cell death response (HR); (**d**) then, trigged PAMP-triggered immunity (PTI) and ETI lead to the accumulation of salicylic acid (SA), jasmonate (JA), ethylene (ET), and abscisic acid (ABA). SA induces the rapid transcriptional activation of a string of resistance (R) genes. Abbreviations: SAR, systemic acquired resistance; RDRs, the RNA-dependent RNA polymerases; SD, *Solanaceae* domain; CC, coiled-coil domain; NB, nucleotide-binding domain; NB-ARC, Apaf-1, R-protein, and CED-4 domain; LRR, leucine-rich repeat domain; CNLs, coiled-coil nucleotide-binding leucine-rich repeat receptors; TIR, Toll/interleukin-1; TNLs, Toll/interleukin-1 nucleotide-binding leucine-rich repeat receptors; R-protein, resistance protein; NSs, nonstructural protein encoded by the S RNA segment; NSm, nonstructural protein encoded by the M RNA segment.

The viral small interfering RNAs (vsiRNAs) profiles derived from the TSWV genome in an infected tomato were analyzed. The vsiRNAs targeted host genes involved in many pathways, including those related to plant–pathogen interactions [99]. Thus, tomatoes, like other plants, undergo several stages of defense and auto-immunity (Figure 5). Viruses are recognized by the plant pattern-recognition receptors (PRRs), that is, the pathogen–associated molecular patterns (PAMPs) [100]. PAMP-triggered immunity (PTI), which is the first line of defense for immune response in plants when pathogens invade plants [88]. However, rapid pathogen effectors can disrupt PTI response. During virus invasion, nucleotide-binding leucine-rich repeat receptors (NLRs) recognize specific pathogen effectors and trigger effector-triggered immunity (ETI) [100,101,102,103]. Plant NLRs are subdivided into CC-NLRs (CNLs) and Toll/interleukin-1 (TIR)-NLRs (TNLs) according to their N-terminal structures [104]. The NLRs can directly or indirectly identify pathogen effectors and trigger a hypersensitive cell death response (HR) to restrict TSWV to the site of infection [47,105,106].

The tomato Sw-5b belongs to CNLs. NSm from TSWV specifically binds to the extended SD domain of the Sw-5b protein, and the switch activates the receptor. Sw-5b is automatically activated (Figure 5c), and HR is triggered; these processes lead to a robust defense response against tospoviruses [33,47,71,107,108]. The phytohormones salicylic acid (SA), jasmonate (JA), ethylene (ET), and abscisic acid (ABA) play significant roles in PTI and ETI and activate the systemic acquired resistance of plants [109,110,111,112]. The SA signaling pathway has a key role in basal defense against the TSWV in tomato plants [38,113]. TSWV infection robustly up-regulates SA synthesis and increases SA-related defenses [114]. SA accumulates in infected areas and then induces the rapid transcriptional activation of a string of resistance (R) genes (Figure 5d) [115]. It further triggers HR. JA-related response in TSWV-infected plants are repressed by SA, and this process mainly occurs downstream of the JA biosynthesis pathway [116]. The up-regulation of ABA-related genes results in the suppression of SA-mediated defense [114,117]. Pathogenesis-related 1 (PR-1) and PR-5 are associated with Sw-7 resistance and might play a major role in resistance against TSWV infection [37,87].

RNA interference (RNAi), as a conserved regulatory function mechanism, plays pivotal roles in gene regulation and defense against invading viruses. Therefore, TSWV-infected tomatoes and other plants have similar resistance mechanisms against the TSWV [99,118]. The RNAse III Dicer-like proteins (DCLs), Argonautes (AGOs), and RNA-dependent RNA polymerases (RDRs), the three main stages of the RNAi pathway, are triggered after TSWV infection [119,120,121]. The formation of viral dsRNA by TSWV is diced by DCLs culminating in the production of 21and 22 nt vsiRNAs from the three RNA segments of the TSWV [118]. One of these vsiRNAs is recruited onto AGOs and loaded into the RNA-induced silencing complex (RISC) [122]. Through the action of a target mRNA with siRNA-sequence complementarity, this vsiRNA facilitates the cleaving of an RNA target into small fragments or inhibits translation (Figure 5a,b) [123,124]. The RDRs of plants used vsiRNA as a template for synthesizing dsRNA and amplifying of silencing [118,121,125,126]. Thus, plants resist invading viruses though their own RNAi immune mechanisms [120,124,127]. RDR1 has a dual function, is involved in SA resistance pathways, and inhibits the RDR6-mediated antiviral RNAi pathway [128]. The virus does not show weakness. The NSs protein, as a silencing suppressor of TSWV, inhibits RISC activity in plants by binding AGOs, and then the suppressor RNAi mechanism of plants (Figure 5a,b). It also suppresses plant resistance [129,130]. In general, the plant RNAi immune mechanism plays a role in resisting the invasion of defenseless external viruses.

## 8. Challenge and Prospects

In the breeding of tomato resistant to the TSWV, utilizing existing resistance genes and screening novel resistance genes is a top priority. These methods are environmentally friendly, economical, and effective in alleviating damage due to TSWV infection. *Sw-7* has been mapped in the ~70 kb genomic region. We believe it will be cloned soon. *Sw-5*, *Sw-6*, and *Sw-7* exhibit specific and different resistance mechanisms for TSWV, and complementary resistance is present between them. Thus, we should use these genes as resistance resources and use the MAS technology in aggregating different resistance genes, which will provide stable and lasting resistance. In the plant immune system, the R gene is an essential defense recognition gene [131]. *Sw-5* is the only class *R* identified in tomato, and the *Sw-7* locus belongs to the *R* gene. A detailed *R* locus physical map was built, and the 368 candidate pathogen recognition genes were found on 12 chromosomes in tomato, including 154 NBS-LRR domain resistance-like genes [70,131,132]. As a marker gene for disease resistance, PR-1 possibly plays a role in resistance to TSWV infection in the *Sw-7* line [37]. We infer that the *R* locus is a readily available resource for screening TSWV resistance genes. TSWV tomato species with high resistance and even completely immune effectiveness have not been found. Therefore, in light of the discovery of novel highly activated TSWV races and increasingly serious spread of TSWV, the collection of resistance resources and the discovery of new resistance genes (including the class of *R* gene) against TSWV by combining mechanical inoculation with TSWV and MAS are essential for tomato resistance breeding and cultivation production.

The plant innate RNAi mechanism has a significant defense against TSWV invasion, particularly in tomatoes carrying *Sw-5* [99,127]. In transgenic plants in tobacco (*Nicotiana tabacum*) plants, expressed N protein can resist TSWV infection [119,133]; it reflects an RNA-mediated defense mechanism [12,134,135,136]. The constructed RNAi-mediated transgenic plants by targeting the *N*, *NSm* and *NSs* genes of TSWV indicated that enhanced tobacco lines resistance [137,138]. Additionally, the dsRNAs targeting the *N* gene by the RNAi-based vaccination approach can protect the *Nicotiana benthamiana* and tomato [139]. As the first layer of defense for immune response, the plant immune system prevents the TSWV invasion through en RNA silencing mechanism [33]. Therefore, the RNAi-mediated technology is feasible for enhancing TSWV resistance and tomato resistance breeding.

The genome-editing technology (GET) has become a common technology in recent years. The clustered regularly interspaced short palindromic repeats (CRISPR)/CRISPR-associated protein-9 nuclease (Cas9) system has been used in improving or mitigating viral disease resistance in plants [140,141]. The CRISPR/Cas9 can efficiently achieve specific site-specific mutations and regulate specific plant immune responses [142]. Strategies to improve plant disease resistance by GET, including, modified *R* gene, transformed *susceptible* (*S*) gene, and targeted degradation of viral genome. The synthetic I2 immune receptor CNL of tomato by CRISPR/Cas9 system demonstrates recognition activity and improves tomato resistance to *Phytophthora infestans*, *Fusarium oxysporum* f. sp. *lycopersici*, and phylogenetically divergent pathogens [143]. Additionally, the integration of NLRs from wild species into cultivated tomato enhances resistance [144]. As a typical *S* gene from rice, *Pi21* has been precisely edited with CRISPR/Cas9 systems, and mutant lines displayed enhanced resistance to blast and bacterial blight in rice [145]. *Francisella. novicida* Cas9 (FnCas9) targets the cucumber mosaic virus (CMV) RNA and induces resistance to RNA viruses, and resistance is stably inheritable [146]. However, given that GET applications in tomato TSWV resistance breeding have not been reported, we should use tomato-suitable CRISPR/Cas9 technology for tomato resistance breeding [147]. The *Sw-5a^S^* from TSWV-susceptible cultivars can be transformed through fixed-site precise editing with CRISPR/Cas9, and Sw-5b-mediated TSWV resistance should be reintroduced for the enhancement of tomato resistance to the TSWV. Additionally, substantial breakthroughs have been achieved in plant engineering of NLRs to defend against pathogens [148]. Huang et al. [149] conducted a stepwise artificial evolution strategy to select Sw-5b mutants, which are effective against resistance-breaking isolates of TSWV, in order to provide a new vision and ideas that resist TSWV by artificial evolution.

## 9. Conclusions

One of the great challenges faced in the tomato breeding program to TSWV-disease resistance is the achievement of a stable and lasting resistance. Despite the unremitting efforts of researchers for more than 30 years for the identification and introgression of resistance, given the continuous mutation of TSWV and the emergence of new isolates, the detection of new germplasm resistant to TSWV disease has been challenging. In this review, we focused on the TSWV-resistant tomato breeding plants, TSWV resistance genes, and linked molecular markers, especially the systematic overview of TSWV disease resistance mechanisms, and in-depth elucidation of the molecular mechanisms of TSWV resistance. Disease breeding has far-reaching significance. In recent years, new breeding strategies have surpassed the classic breeding methods by systematic summary of the disease-resistance mechanism of plants against TSWV. The use of new breeding strategies, such as RNA silencing mechanism, targeted gene editing, and NLR artificial evolution, to achieve plants resistant to TSWV infection is a new opportunity in tomato breeding, particularly in disease resistance.

## Figures and Tables

**Table 1:** Tomato resources with TSWV resistance genes.

Genotype/Lines/Cultivars	Material Source	Resistance Gene	Notes	References
Porter’s	*L. pimpinellifolium*	*Sw-1a*, *sw-2*, *sw-3*, *sw-4*	Resistance	[44]
Pearl Harbor	*L. pimpinellifolium*	*Sw-1a*, *sw-4*	Resistance	[45]
PI 732293-2V	*L. pimpinellifolium*		Resistance	[46]
LA0411	*L. pimpinellifolium*	*Sw-5b*	Resistance	[47]
PI 128657	*L. peruvianum*	*Sw-1*	Resistance	[48]
LA-444-1	*L. peruvianum*		Resistance	[46]
Viradora	*L. peruvianum*	*Sw-5*	Resistance	[49]
Stevens	*L. peruvianum*	*Sw-5a*, *Sw-5b*, *Sw-5c*, *Sw-5d*, *Sw-5e*	Resistance	[50,51,52]
LA0370, LA0445, LA0446, LA2581, LA4445	*L. peruvianum*	*Sw-5b*	Resistance	[47]
PE-18/UPV-1	*L. peruvianum*	*Sw-5*	Resistance	[53]
RDD	*L. peruvianum*	*Sw-5*	Resistance	[53]
LA2744	*Solanum peruvianum*	*Sw-5*	Resistance	[36]
PI-126928, PI 126944, LA 444-1, LA 371	*L. peruvianum*		Resistance	[54]
PI-126935, CIAPAN 16, PE-18, CIAPAN 17	*L. peruvianum*		Resistance	[42]
TSWV. CNPH-201, 374LA-111, 372, 385, 441-1, 1113-1PI 126930, PI 126946, PI 128659, PI 128660, PI 129146	*L. peruvianum*		Resistance	[48,55]
UPV-101	*L. peruvianum*		Resistance	[56]
PI-126944	*L. peruvianum*		Resistance	[56]
Rey de los Tempranos	*L. esculentum*	*Sw-1b*, *sw-2*, *sw-3*, *sw-4*	Resistance	[44,57]
Manzana	*L. esculentum*	*Sw-1b*, *sw-3*	Resistance	[45]
Anahu	*L. esculentum*		Resistance	[58]
NC 58S, NC 123S, NC 127S, NC 132S	*L. esculentum*	*Sw-5*	Resistance	[59]
Y118 (Fla 925-2)	*L. esculentum*		Resistance	[43]
PE-18/UPV-1	*L. esculentum*		Resistance	[45]
‘Moneymaker’	*L. esculentum*	*Sw-5f*	Susceptible	[60]
LA1204	*L. esculentum*	*Sw-5b*	Resistance	[47]
PE-28_1_/UPV-32	*L. esculentum*	*Sw-6*	Resistance	[45]
PI-127826	*L. hirsutum*		Resistance lost	[46,48]
LA-1353	*L. hirsutum*		Resistance	[46]
LA-1223	*L. hirsutum*		Resistance	[46]
PI-134417	*L. hirsutum*			[46]
LA0716, LA0751	*L. pennellii*	*Sw-5b*	Resistance	[36,47]
LA2113	*L. neorickii*	*Sw-5b*	Resistance	[47]
LA1358, LA1365, LA1777, LA2809	*L. huaylasense*	*Sw-5b*	Resistance	[36,47]
LA0747	*L. galapagense*	*Sw-5b*	Resistance	[47]
LA0103, LA1331, LA1722	*L. corneliomuelleri*	*Sw-5b*	Resistance	[47]
LA1407, LA0927	*L. cheesmaniae*	*Sw-5b*	Resistance	[47]
LA1350	*L. arcanum*	*Sw-5b*	Resistance	[47]
LA1028	*Solanum chmielewski*	*Sw-5*	Resistance	[36]
LA4110	*Solanum* *. sitiens*	*Sw-5*	Resistance	[36]
LA1930	*L. chilense*	*Sw-5b*	Resistance	[47]
LA-2931	*L. chilense*		Resistance	[61]
LA 130, LA 2753	*L. chilense*		Resistance	[54]
LA 1938	*L. chilense*	*Sw-7*	Resistance	[43]

**Table 2:** Molecular markers associated with TSWV-resistant in tomato.

Markers Name	Markers	Gene	Fragment Size (bp)		Primer Sequence (Forward, F; Reverse, R)	Reference
			Resistant	Susceptible		
#72	RAPD	*Sw-5*	~2200	-	F: 5′-GAGCACGGGA-3′	[62]
SCAR421	SCAR	*Sw-5*	940	900	F: 5′-GACTTGTTGCCATAGGTTCC-3′ R: 5′-GCCCACCCCGAAGTTAATCC-3′	[89]
CT220	AFLP	*Sw-5*	~400	~250	F: 5′-GAAGCCCTGCTTTTGTTTCGATCC-3′ R: 5′-CAACGATGGTACCGATGGATCGAA-3′	[69]
SCr2	SCAR	*Sw-5*	400	-	F: 5′-CTGGGTGAGTCTTGACATTT-3′ R: 5′-CTGGGTGAGTACATCAGATT-3′	[90]
S12	RAPD	*Sw-5*	~400	-	F: 5′-CTGGGTGAGT-3′	[91]
G5	RAPD	*Sw-5*	~700	-	F: 5′-CTGAGACGGA-3′	[91]
K16	RAPD	*Sw-5*	~1400	-	F: 5′-GAGCGTCGAA-3′	[91]
ZUP64	CAPS	*Sw-5*	213	94	F: 5′-AAGCCGAATTATCTGTCAAC-3′ R: 5′-GTTCCTGACCATTACAAAAGTAC-3′	[92]
Sw-5b-LRR	CAPS	*Sw-5b*	305	94	F: 5′-TCTTATATTGTGGAGTTTTTGTCG-3′ R: 5′-TCCACCCTATCAAATCCAAC-3′	[92]
Sw-5-2	In-Del	*Sw-5*	574	510/464	F: 5′-AATTAGGTTCTTGAAGCCCATCT-3′ R: 5′-TTCCGCATCAGCCAATAGTGT-3′	[93]
Sw5-f2/r2	SNP	*Sw-5b*	541	-	F: 5′-CGGAACCTGTAACTTGACTG-3′ R: 5′-GAGCTCTCATCCATTTTCCG-3′	[94]
NCSw-007	CAPS	*Sw-5*	480	240	F: 5′-GTTGCTAACTCGACTCGTTC-3′ R: 5′-TCACTCACGTCCTATTGACA-3′	[95]
NCSw-011	CAPS	*Sw-5a*	430	600	F: 5′-TATCATCCTCATACCCCTTG-3′ R: 5′-GGATTTTCTCATCATCTCCA-3′	[95]
NCSw-003	SCAR	*Sw-5*	680	600	F: 5′-TCTCGTTATCCAATTTCACC-3′ R: 5′-GCAATTTTGTTTCTTGGTCT-3′	[95]
NCSw-012	SCAR	*Sw-5b*	-	1000	F: 5′-ATGGTCAACTCGATCAGAAC-3′ R: 5′-TTTGGTGAGGATCTGATTTC-3′	[95]
Sw5b-SNP	SNP	*Sw-5b*	100	-	F: 5′-TTATTGTTTCTCGCTTTGATGTTCG-3′ R: 5′-GAACCTGTAACTTGACTGAAAATATC-3′	[96]
KASP	KASP	*Sw-5*	-	-	F_C: 5′-AATAATTAAAACAAACATTRTAGGTATGGTAAC-3′ F_A: 5′-AATAATTAAAACAAACATTRTAGGTATGGTAAA-3′ R: 5′-GTGTTTGTTTAACACTYTGTTTGAATCATT-3′	[97]
CT99	RFLP	*Sw-7*	880	850	F: 5′-GTCCCGGTGACATACTTACTG-3′ R: 5′-AGATTCTGTGTTGGAGGTGAGT-3′	[98]
SSR20	SSR	*Sw-7*	157	148	F: 5′-GAGGACGACAACAACAACGA-3′ R: 5′-GACATGCCACTTAGATCCACAA-3′	[66]
-	SCAR	*Sw-7*	285	241	F: 5′-GAAGGTGCCGACGGTGTA-3′ R: 5′-AGGAATCAAGGTAAACCACCA-3′	[37]

Note: RAPD (random amplified polymorphic DNA); CAPS (cleaved amplified polymorphic sequence); InDel (insertion-deletion); SNP (single-nucleotide polymorphism); SCAR (sequence characterized amplified regions); RFLP (restriction fragment length polymorphism); KASP (kompetitive allele-specific PCR). The dashes indicate no fragment size or non-reported; the wavy line represents approximately fragment size.

## Data Availability

All the data is contained within the article.

## Abbreviations

TSWV	Tomato spotted wilt virus
L RNA	Large RNA
M RNA	Medium RNA
S RNA	Small RNA
RdRp	RNA-dependent RNA polymerase
G_n_G_c_	Glycoprotein precursors
NSm	Viral non-structural proteins
NSs	Non-structural proteins
N	Nucleocapsid proteins
TB2	TSWV strains tip blight 2
N1	TSWV strains necrotic
R1	TSWV strains ringspot
TB3	TSWV strains tip blight 3
TCSV	Tomato chlorotic spot virus
TZSV	Tomato zonate spot virus
GRSV	Groundnut ring spot virus
INSV	Impatiens necrotic spot virus
*R*	Resistance gene family
SD	*Solanaceae* domain
CC	Coiled-coil domain
NB-ARC	Nucleotide binding-adapter shared by APAF-1, R proteins and a CED-4
LRR	Leucine-rich repeat domain
APAF-1	Apoptotic protease-activating factor-1
PR-1	Pathogenesis-related protein 1
PR-5	Pathogenesis-related protein 5
MAS	Marker-assisted selection
RAPD	Random amplified polymorphic DNA
CAPS	Cleaved amplified polymorphic sequence
InDel	Insertion-deletion
SNP	Single-nucleotide polymorphism
SCAR	Sequence characterized amplified regions
RFLP	Restriction fragment length polymorphism
KASP	Kompetitive Allele-Specific PCR
vsiRNAs	Viral small interfering RNAs
PRRs	Plant pattern-recognition receptors
PAMPs	Pathogen–associated molecular patterns
PTI	PAMP-triggered immunity
NLRs	Nucleotide-binding leucine-rich repeat receptors
ETI	Trigger effector-triggered immunity
CNLs	CC-NLRs
TNLs	Toll/interleukin-1 (TIR)-NLRs
HR	Hypersensitive cell death response
RNAi	RNA interference
vds RNAs	Viral dsRNA
DCLs	RNAse III Dicer-like proteins
RDRs	RNA-dependent RNA polymerases
AGO	Argonautes
RISC	RNA-induced silencing complex
SA	Salicylic acid
JA	Jasmonate
ET	Ethylene
ABA	Abscisic acid
SAR	Systemic acquired resistance
GET	Genome-editing technology
CRISPR/Cas9	Clustered regularly interspaced short palindromic repeats (CRISPR)/CRISPR-associated protein-9 nuclease (Cas9) system
*S*	*Susceptible* gene
CMV	Cucumber mosaic virus
FnCas9	*Francisella. novicida* Cas9
